# Remodeling of intracellular architecture during SARS-CoV-2 infection of human endothelium

**DOI:** 10.1038/s41598-024-80351-z

**Published:** 2024-11-30

**Authors:** Agata Kubisiak, Agnieszka Dabrowska, Pawel Botwina, Patrycja Twardawa, Damian Kloska, Tomasz Kołodziej, Zenon Rajfur, Krzysztof Pyrc, Marta Targosz-Korecka

**Affiliations:** 1 Department of Physics of Nanostructures and Nanotechnology, Jagiellonian University, Faculty of Physics, Astronomy and Applied Computer Science, M. Smoluchowski Institute of Physics, Kraków, Poland; 2 Doctoral School of Exact and Natural Sciences, Jagiellonian University, Kraków, Poland; 3 Malopolska Centre of Biotechnology, Virogenetics Laboratory of Virology, Jagiellonian University, Kraków, Poland; 4 Department of Medical Biotechnology, Jagiellonian University, Faculty of Biochemistry, Biophysics and Biotechnology, Kraków, Poland; 5 Selvita Services, Kraków, Poland; 6 Department of Pharmaceutical Biophysics, Faculty of Pharmacy, Jagiellonian University Medical College, Kraków, Poland; 7 Department of Molecular and Interfacial Biophysics, Jagiellonian University, Faculty of Physics, Astronomy and Applied Computer Science, M. Smoluchowski Institute of Physics, Kraków, Poland

**Keywords:** SARS-COV-2, COVID-19, Endothelial cells, Variants, Elasticity, AFM, Biophysics, Cell biology, Microbiology

## Abstract

Clinical data indicate that COVID-19 causes cardiovascular complications, regardless of the severity of the disease. In this work, we have shown that SARS-CoV-2 infection causes vascular dysfunction due to the modification of endothelial cell elasticity. We used human pulmonary endothelial cells (HPAECs) expressing the ACE2 receptor as a model of the endothelium. This system mimics in vivo conditions, as it allows virus entry but not replication. As a reference, we used A549 epithelial cells, a well-described model that supports productive replication of SARS-CoV-2. We show that the infection of HPAECs results in loss of cell elasticity, which correlates with increased polymerization of actin filaments and induction of the inflammatory response. On the contrary, A549 epithelial cells supporting viral replication showed increased elasticity. We also showed that the endothelial cell elasticity were impaired after infection with Alpha, Beta and Delta variants. Consequently, we believe that nonproductive SARS-CoV-2 infection associated with loss of the endothelium elasticity may be clinically relevant and result in dysfunction and damage to this tissue.

## Introduction

Severe acute respiratory syndrome coronavirus 2 (SARS-CoV-2) that causes coronavirus disease 2019 (COVID-19) promoted the pandemic not seen after the Spanish flu in the beginning of previous century. Extremely high number of cases created a convenient platform for virus evolution and resulted in the rapid emergence of several genetic variants of the SARS-CoV-2. The first three named Alpha (B.1.1.7), Beta (B.1.351), and Delta (B.1.617.2), although they have the same origin, differ in transmissibility and/or severity of the associated disease^1,2^.

The available data unquestionably point to the significant role of the endothelium in the development of the severe course of COVID-19 and the long COVID^3–5^. In most cases, endothelial dysfunction is described as an abnormal cellular phenotype in which vascular balance shifts to vasoconstriction and inflammation. The clinical signs of endotheliitis and vasculitis are reported as post-infection complications that may appear in a wide range of organs, including the kidney, heart, small intestine, lung, and skin^6^. This further supports the observation that COVID-19 is a systemic disease affecting the endothelial compartment^7–9^. However, the biomechanical understanding of this process remains unclear^9,10^.

A distinction is made between direct and indirect causes of the development of endothelial dysfunction. The former is related to the direct consequences of infection, while the latter is the result of endothelial exposure to cytokines (cytokine storm)^11^. Local and systemic inflammation that characterizes COVID-19 activates and damages the endothelium, resulting in an elevation of von Willebrand factor (vWF) in the blood, making the vasculature more susceptible to thrombotic events^12–15^. However, the tissue is also considered the infection site. In postmortem tissue evaluation, Varga^16^ and others^17,18^ identified viral content in blood vessels and blood. A report by Jacobs et al.^19^ published in 2022 refers to the clinical study that proves the occurrence of viral proteins in the plasma of patients with COVID-19. The authors indicate the correlation of viremia with disease severity, its outcome, and specific inflammatory biomarkers.

While virus particles and virus RNA have been found in the blood and endothelial tissues and we know that the SARS-CoV-2 virus can cause abortive infection of the human endothelium, the consequences of the interaction between virus and endothelium in the clinic remain to be elucidated^20^. Some autopsy-based studies following COVID-19 have shown multifocal vascular damage, as well as activation of endothelial cells^21^. Furthermore, among the etiological factors of the observed endothelial dysfunction, pro-inflammatory cytokines produced during COVID-19 were reported. Buzhdygan et al.^22^ have shown that the SARS-CoV-2 spike protein promotes the loss of integrity of the endothelial barrier and increases endothelial inflammation and permeability due to modulation of the renin-angiotensin pathway, potentially affecting the function of the blood–brain barrier. Similarly, Rhea et al.^23^ show that virus-induced structural remodeling of the endothelial tissue results in increased permeability of the blood–brain barrier to the virus. It is also known that the spike protein, which protrudes from the surface of the virus particle, interacts with the angiotensin-converting enzyme 2 (ACE2) protein, which acts as a cellular receptor^24^. Moreover, our recent study^25^ showed that ACE2 is present on the surface of HPAEC cells, hence these cells should become permissive for SARS-CoV-2, however, for efficient infection several other factors such as TMPRSS2 are required. Importantly, an increasing number of scientific reports also indicate the potential role of integrins as SARS-CoV-2 co-receptors in the endothelium^26^.

In this work, we hypothesized that endothelial nanomechanics play an important role in the initiation and progression of endothelial dysfunction during SARS-CoV-2 infection. Nanomechanical properties of endothelial cells such as cellular elasticity constitute an important part of the endothelial phenotype and their alteration is a starting point of endothelial dysfunction development^27–29^. As we have shown in previous works, the early response of endothelial cells to pathological factors (e.g., proinflammatory cytokines, high glucose) is associated with increased cell stiffness and actin cytoskeleton remodeling, resulting in decreases in nitric oxide (NO) production and activation of the inflammatory pathway^30–32^. Consequently, nanomechanical changes occurring in endothelial cells contribute to the development of vascular dysfunction, atherosclerosis, and hypertension and ultimately cause serious systemic complications^29^.

In our study, we evaluate the nanomechanical response of human pulmonary artery endothelial cells (HPAECs) and A549 epithelial cell line (as a cell model standardly used in research on coronaviruses) expressing ACE2 and TMPRSS2 (A549^+/+^) infected with SARS-CoV-2 highlighting the important role of virus variability (Wuhan, Alpha, Beta, Delta) in the course of the nanomechanical changes. We have analyzed cell topography correlated with cell elasticity maps by using the atomic force microscopy (AFM) method completed by fluorescence microscopy and quantitative PCR coupled with reverse transcription (RT-qPCR) analysis.

## Results

### SARS-CoV-2 induces abortive infection in HPAEC cells

**Fig. 1:**
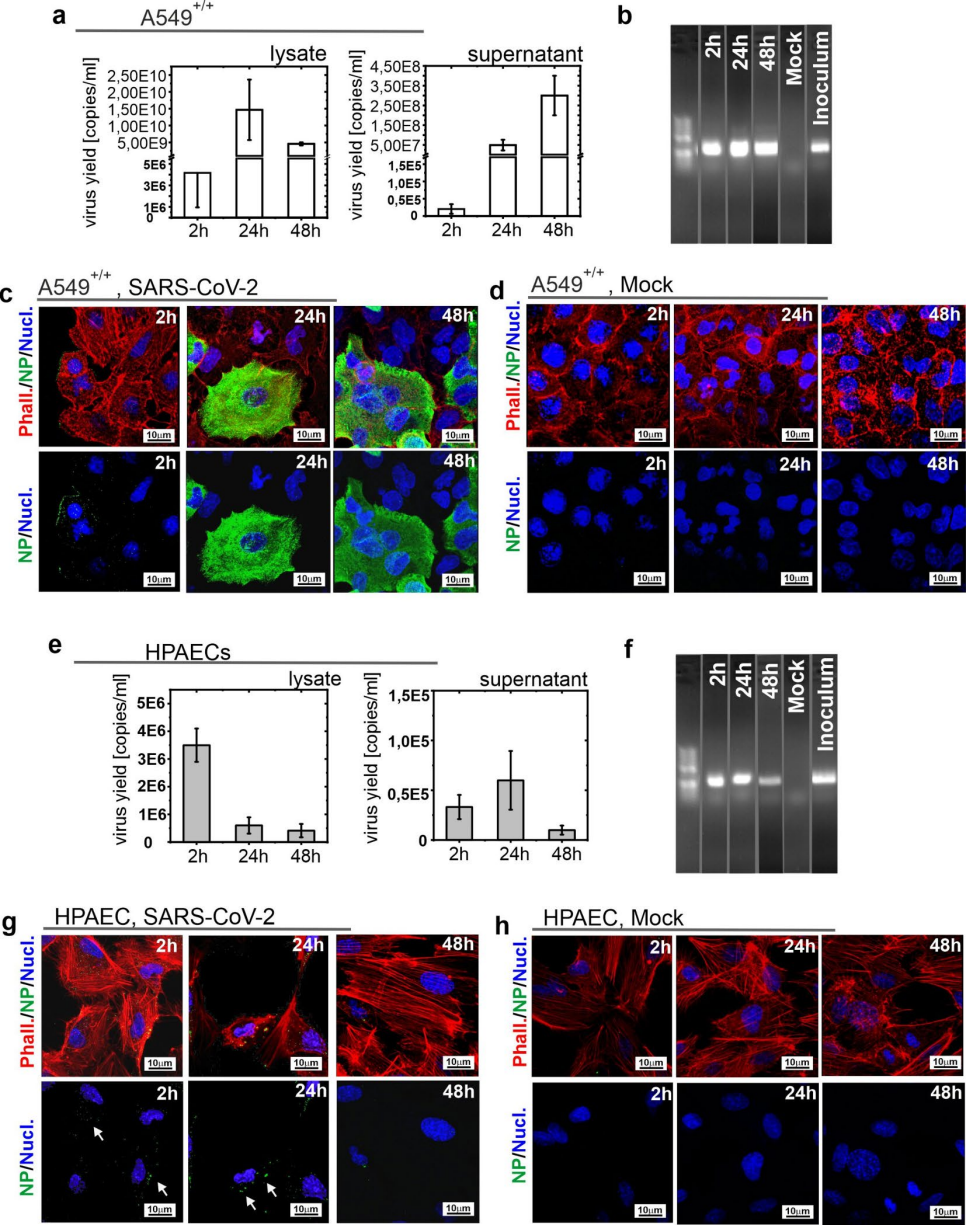
Assessment of SARS-CoV-2 (Wuhan) replication in A549^+/+^ cells and HPAECs. (**a**) The graphs show the RT-qPCR results indicating the virus yield in the SARS-CoV-2 infected (5000 TCID_50_/ml) A549^+/+^ cells (lysates and supernatants) collected at 2, 24 and 48 h p.i.. The results are presented as average values with standard deviations (error bars). (**b**) PCR analysis of subgenomic mRNA present in A549^+/+^ cells obtained in cells infected with SARS-CoV-2 at 5000 TCID_50_/ml and collected at 2, 24, and 48 h p.i. Mock-inoculated cells were used as a negative control, while the inoculum was used as a positive control. The PCR products were visible around 250 bp as expected. (**c**, **d**) Representative immunofluorescence images of actin filaments, nucleocapsid protein (NP), and nucleus were obtained for A549^+/+^ cells infected with SARS-CoV-2 (**c**) or mock-inoculated (**d**). Merged panels (upper row) and separate (bottom row). Appropriate analysis as for A549^+/+^ was performed for HPAECs cells infected with the SARS-CoV-2 virus i.e.: (**e**) virus yield in the cells lysates and supernatants, (**f**) PCR analysis of sg mRNA present in HPAECs (**g**, **h**) immunofluorescence images of actin filaments, nucleocapsid protein (NP) and nucleus obtained for infected HPAECs (**g**) and mock-inoculated (**h**). Actin filaments—red, nucleocapsid protein—green, nucleus—blue. Experiments were performed in at least two repetitions, each in duplicate. Fluorescence microscope images were collected on the Zeiss LSM 710 using 40×/1.3 oil objective.

A549^+/+^ and HPAEC cells were infected with the SARS-CoV-2 virus. At 2, 24, and 48 h post infection (p.i.), the supernatant and cells were collected for RT-qPCR and subgenomic viral mRNA (sg mRNA) analysis, and the cells were fixed. Detection of sg mRNA was carried out to confirm active viral replication, because once the virus enters cells, replication of the viral genome and production of sg mRNA begins. Human lung adenocarcinoma A549^+/+^ cells are a permissive for SARS-CoV-2 virus infection and were used as a positive control. We confirmed the efficient replication in A549^+/+^ cells by RNA quantification (Fig. 1a), the presence of viral sg mRNA (Fig. 1b), and by the nucleocapsid protein detection (Fig. 1c,d). The same techniques were used to confirm infection in HPAEC cells; SARS-CoV-2 virus does not replicate productively in the cells tested, as indicated by RT-qPCR analysis (Fig. 1e**)**; however, the presence of sg mRNA indicates that the genome replication occurs (Fig. 1f). In addition, using an immunostaining method, virus particles (indicated by white arrows) were labeled after 2 and 24 h p.i. (Fig. 1g,h). The results consistently indicate the abortive infection of SARS-CoV-2 in HPAEC cells.

### Impact of SARS-CoV-2 infection on the nanomechanical properties of A549^+/+^ cells and HPAECs

**Fig. 2:**
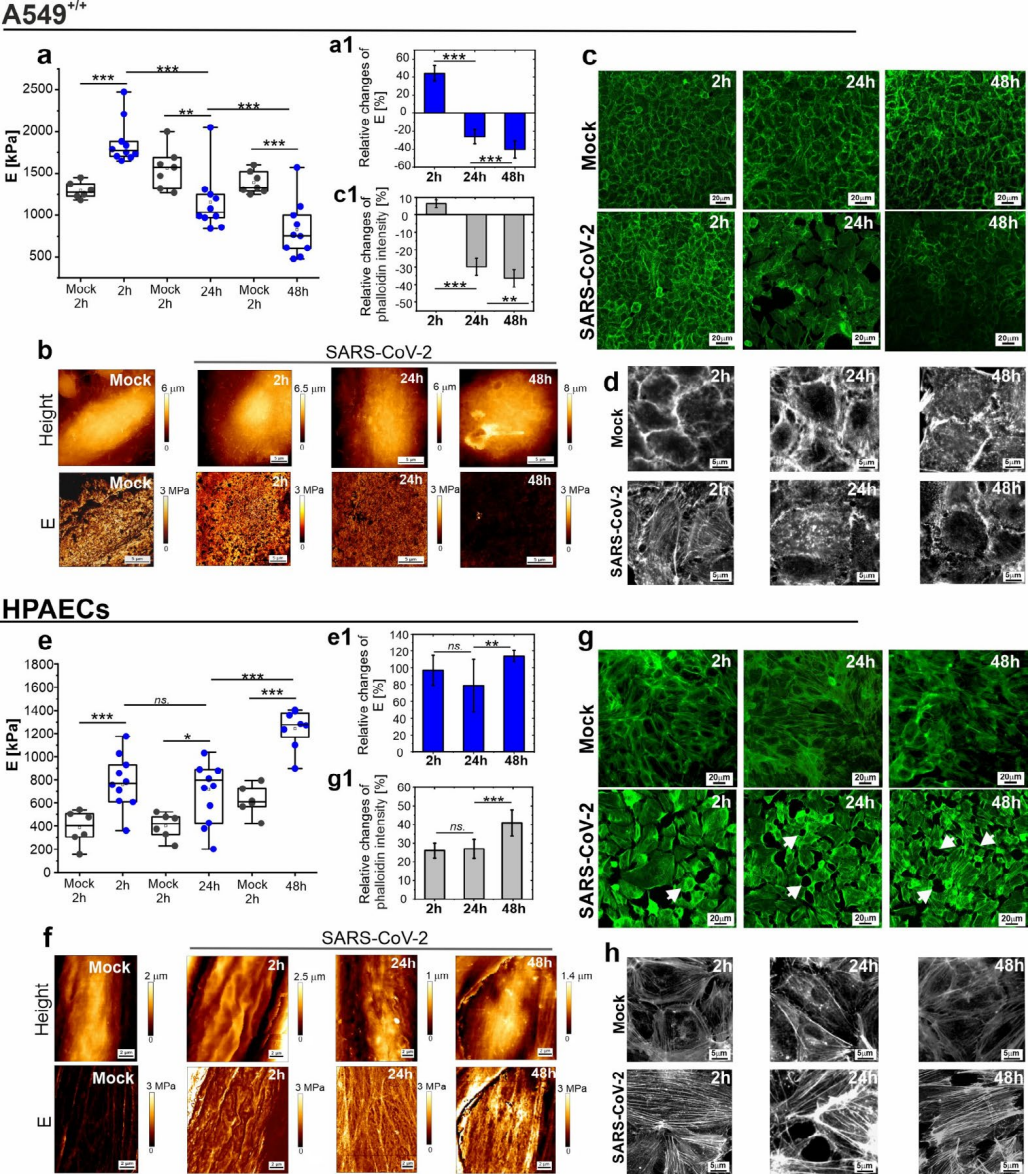
Post-infection changes of cellular elasticity and cytoskeleton structure. (**a**–**h**) The nanomechanical analysis of A549^+/+^ cells infected with SARS-CoV-2 (Wuhan): (**a**) Box-plots depict the quantitative analysis of elastic modulus calculated for A549^+/+^ cells after 2, 24, and 48 h p.i. and reference mock-inoculated cells. All measurements were performed using fixed cells. Each point in the box plot represents the mean value of E calculated for a single cell. A1) Graph represents the relative changes of E with respect to mock. (**b**) Representative AFM maps of height (top row) and E (bottom row) were taken for A549^+/+^ cells infected with SARS-CoV-2 and mock-inoculated (representative images for 24 h p.i.). (**c**) Representative fluorescence images of A549^+/+^ stained for F-actin. The top rows represent mock images, and the bottom row represents the images taken for infected A549^+/+^. (**c1**) Quantification of phalloidin intensity showed as relative changes to mock. (**d**) Representative fluorescence images of A549^+/+^ stained for F-actin in grey scale and higher magnification (40×). The top rows represent mock images, and the bottom row represents the images taken for infected A549^+/+^. Appropriate nanomechanical analysis obtained for HPAECs are shown in (**e**–**h**) panel. (**e**) The quantitative analysis of elastic modulus for infected and mock HPAECs. (**e1**) Graph of the relative changes of E to mock. (**f**) Representative AFM maps of height (top row) and E (bottom row) taken for HPAECs. (**g**) Representative fluorescence images of HPAECs stained for F-actin. The top row represents mock images and the bottom row represents the images taken for HPAECs after 2, 24, and 48 h p.i. The white arrows mark the characteristic gaps formation in the endothelial layer. (**g1**) Quantification of phalloidin intensity presented as relative changes to mock cells. (**h**) Representative fluorescence images of HPAEC stained for F-actin in grey scale and higher magnification (40×). The top rows represent mock images, and the bottom row represents the images taken for infected HPAEC. F-actin—green. All measurements were made for fixed cells. Experiments were performed in at least two repetitions. Statistics: p values were determined by one-way ANOVA followed by Tukey’s post-hoc test, with significance marked as (*): p < 0.05; (**): p < 5E − 3; (***): p < 5E − 6. The Fig. was created with OriginPro2022.

Differences in the course of infection for A549^+/+^ cells and HPAECs prompted us to perform nanomechanical measurements. Our aim was to check whether changes in elastic modulus (*E*) were observed despite the lack of effective infection of HPAECs. An increase in E indicates that the cells are losing their elasticity, while a decrease in E indicates an increased elasticity of the cells compared to the reference sample. Figure 2 shows the results of the AFM nanomechanical measurements and actin cytoskeleton imaging obtained for A549^+/+^ and HPAECs infected with SARS-CoV-2 in the following time points: 2, 24, and 48 h.

A549^+/+^ cells showed a time-dependent, biphasic change of the *E* values. After 2 h p.i., the values of *E* significantly increased for all measured cells in comparison to the mock sample, as shown on Fig. 2a. For the subsequent timepoints (24 h and 48 h), a significant decrease in *E* value was observed. The relative changes plot shown in Fig. 2a1 indicates the percentage change in the *E* relative to mock. For 2 h p.i. times, the *E* increases by 44% compared to mock, while for the remaining times it decreases, reaching a drop of 42% after 48 h p.i. The quantitative analysis of *E* was supported by qualitative data visualization in a form of 2D AFM images of cells morphology correlated with distribution of *E* (elasticity maps), as showed in Fig. 2b. By maintaining a constant range of *E* values for all elasticity maps, a significant reduction of this parameter is shown with increasing time of p.i. and it is correlating with gradual injury to the cell structure. Observed nanomechanical changes are strengthened by fluorescence images of F-actin structure in infected cells (Fig. 2c) as well as quantitative analysis of fluorescence intensity of phalloidin (Fig. 2c1). All together indicate progressive damage of the A549^+/+^ cells after infection with SARS-CoV-2 occurring parallelly with a significant change in elasticity, and F-actin content based on viral replication in infected cells, as well as cytopathic effect observations manifested by, among other things, reduced cell confluence and syncytium formation^33^.

HPAECs infected with SARS-CoV-2 also showed significant changes in cell elasticity and structure, which correlated with the remodeling of the actin cytoskeleton. At first, similar to the infected A549^+/+^ cells, significant decrease in cellular elasticity (96% relative to mock) has been observed at 2 h p.i. Additionally, for this timepoint, the AFM image and elasticity map (Fig. 2f) depict rigid membrane ripples on the cell surface, which leads to the conclusion that after infection there are changes in the perimembrane structures of the cell. After 24 h p.i, the values of *E* measured for subsequent cells did not change significantly relative to values obtained for 2 h p.i, as depicted in Fig. 2e. However, in relation to mock, the infected HPAECs are significantly less elastic. The AFM image shows some structural changes in the cell membrane, and the elasticity map depicts a highly crosslinked actin network under the cell membrane. Observed changes indicate that infection with SARS-CoV-2 involves the cytoskeletal remodeling in HPAECs. This hypothesis is confirmed by result noted after 48 h p.i. For this time-point, further significant increase of *E* value was proven by quantitative analysis (Fig. 2e,e1) and qualitative analysis of AFM images (Fig. 2f) showing a rigid cortex covering the cell body. The fluorescence analysis depicts in Fig. 2g for 2 h p.i. validate the AFM results, showing increase F-actin content in HPAECs infected with SARS-CoV-2. Moreover, the graph of relative changes (**Fig. 2e1**) highlights changes in relation to mock, indicating that the most significant increase of *E* valueoccurs after 48 h p.i. Moreover, the presented decrease in elasticity of HPAECs as well as the reorganization of the actin cytoskeleton leads to changes in endothelial layer structure, Fig. 2g (bottom row), manifested by the loss of intercellular connections and the formation of characteristic gaps in the endothelial structure (oval spaces formed between cells marked with white arrows), suggesting an increase in the permeability of the endothelial layer. An increase in endothelial permeability and an increase in endothelial stiffness directly indicate the development of endothelial dysfunction caused by the SARS-CoV-2 virus.

To address the question of the mechanism of the post infection cytoskeleton remodeling, we focused on RhoA proteins and vimentin playing important regulatory and transport roles in both epithelial and endothelial cells.

**Fig. 3:**
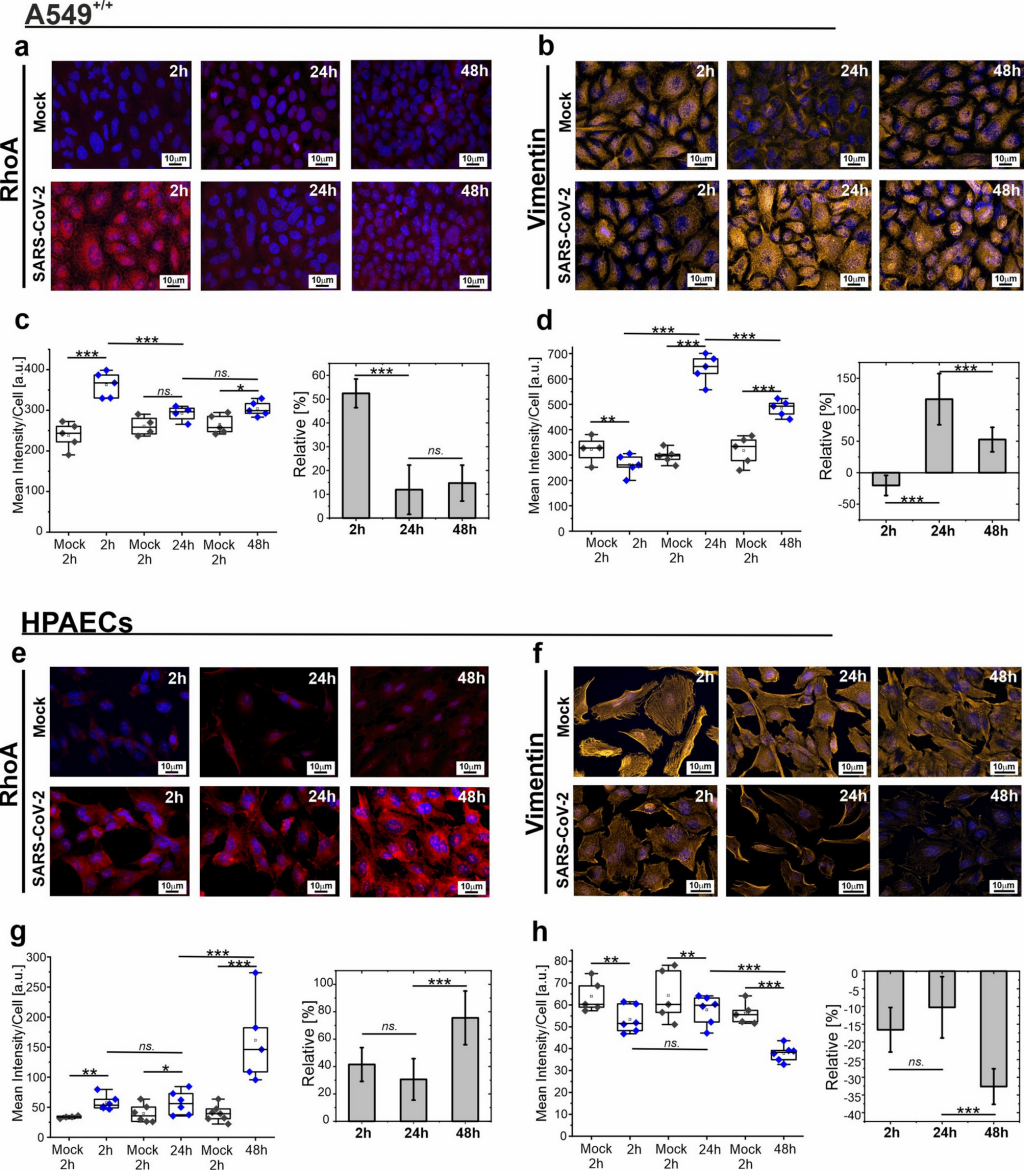
Anti-correlation between the RhoA and vimentin expression in SARS-CoV-2 (B.1.13 variant) infected cells. (**a**) Representative fluorescence images of A549^+/+^ cells stained for a) RhoA protein and (**b**) vimentin taken for subsequent post infection times: 2 h, 24 h and 48 h. (**c**) Quantification of RhoA staining intensity in A549^+/+^ presented as box-plot of mean intensity values per image normalized to the number of cells (left) and relative changes of intensity to mock (right). (**d**) Quantification of vimentin staining intensity presented as box-plot of mean intensity values (left) and relative changes of intensity to mock (right). The results obtained for HPAECs are shown in (**e**–**h**) panel. (**e**) Representative fluorescence images of RhoA and (**f**) vimentin and corresponding (**g**, **h**) quantification of fluorescence intensity for (**g**) RhoA and (**h**) vimentin. Rho-A—red, vimentin—yellow. Experiments were performed in at least two repetitions, each in duplicate. Statistics: p values were determined by one-way ANOVA followed by Tukey’s’ post-hoc test, prepared in Origin software. The statistical significance was marked as: (*): p < 0.05, (**): p < 5E − 3, (***): p < 5E − 6.

Figure 3 shows the results of qualitative and quantitative analysis of fluorescence images obtained for RhoA protein and vimentin in A549^+/+^ cells and HPAECs. Images were taken after 2 h, 24 h, and 48 h post infection and, as a reference, images were taken for mock for these same times points. In the case of A549^+/+^ cells (Fig. 3a,c) it was noticed an initial increase in the RhoA protein content (2 h p.i.). However, for the following 24 h and 48 h, a significant decrease of RhoA protein content was observed. Interestingly, an increase in the amount of RhoA protein is accompanied by a decrease in the amount of vimentin and, conversely, a decrease in the amount of RhoA protein is associated with an increase in the amount of vimentin, as shown in Fig. 3b,d.

A similar effect was noted for HPAECs. The results presented in Fig. 3e–h indicate an increase in the amount of RhoA protein at subsequent p.i. time points and, in parallel, for the same p.i. time points, the vimentin signal is decreasing. Obtained results indicate the important role of RhoA and vimentin in the course of response of epithelial and endothelial cells to SARS-CoV-2 infection. Moreover, changes in RhoA expression associated with the formation of characteristic gaps in the cell layer are consistent with literature data ^34,35^ and our work on the activation of endothelium by the cytokine TNF-a^36^. Additionally, in the work by Claesson-Welsh et al.^37^ and Adamson et al.^38^ the authors indicate that the increase in RhoA leads to the formation of radial stress fibers and increased contractility and permeability of endothelial monolayers.

### Intracellular changes during SARS-CoV-2 infection in HPAECs

However, in this work, we ask about the impact of the virus on the nanomechanical properties of HPAECs and linking these changes with the endothelial cell’s phenotype.

**Fig. 4:**
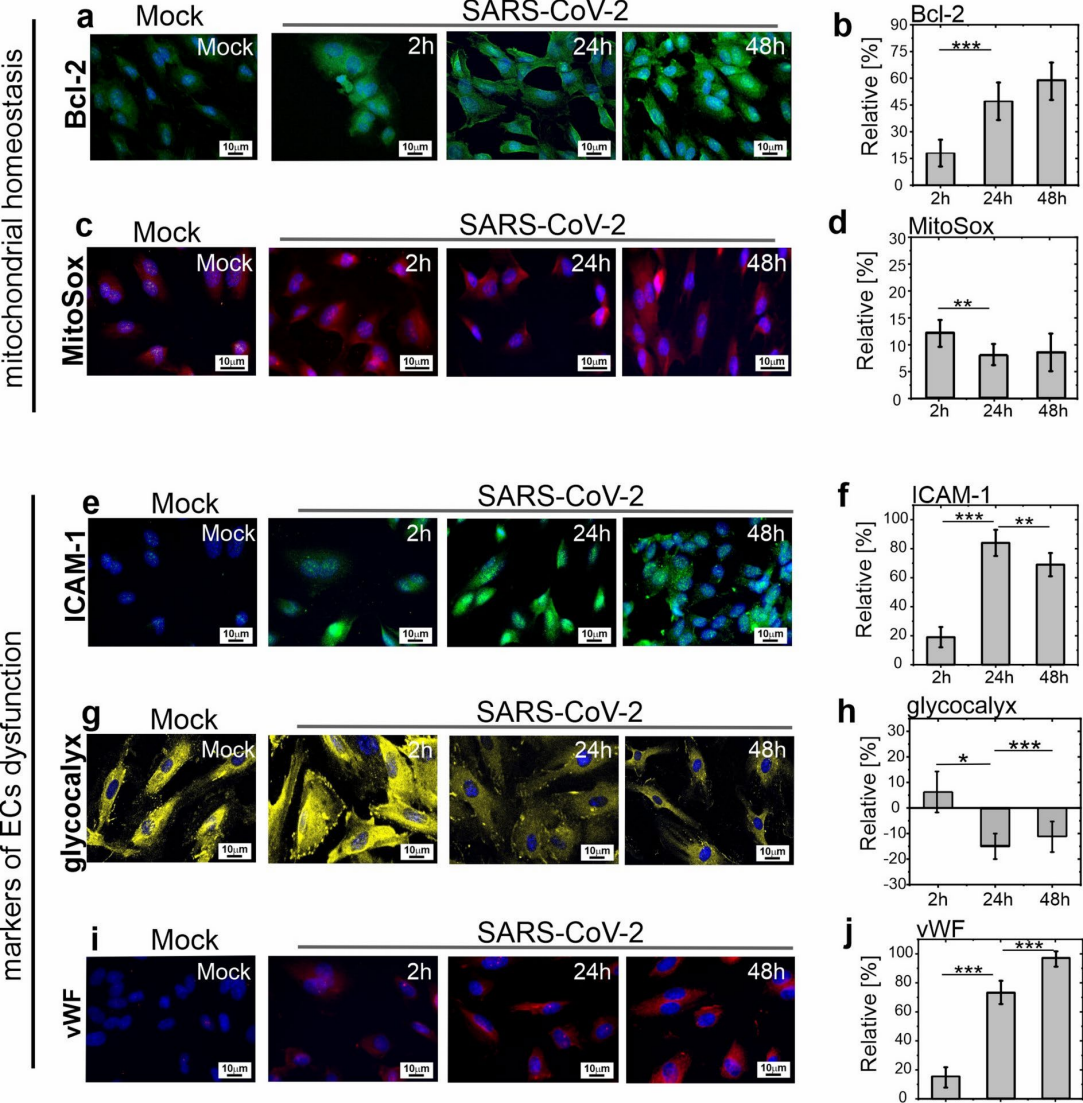
Markers of HPAECs dysfunction. *Mitochondrial homeostasis* panel represents Bcl-2 protein and MitoSox analysis performed for HPAECs infected with SARS-CoV-2 (Wuhan). (**a**, **c**) Representative images of Bcl-2 (**a**) and MitoSox (**c**). Mock image was taken for 24 h post inoculation. (**b**, **d**) Quantitative analysis of the fluorescence intensity. Data shown as a relative value to the reference measurements, i.e. ock 2 h, 24 h, and 48 h. Mock image was taken for 24 h post inoculation. Bcl-2-green; MitoSOX—red. *Markers of ECs dysfunction* panel shows the post-infection analysis of ICAM-1 molecule, glycocalyx layer (heparan sulfate, HS), and vWF performed for HPAECs. Fluorescent images of ICAM-1 molecule (**e**), glycocalyx coverage (**g**), and vWF (**i**) were taken for 2 h, 24 h, and 48 h post infection times. Depicted mock image was taken for 24 h post inoculation. Relative changes of fluorescence intensity of ICAM-1 (**f**), glycocalyx (**h**), and vWF (**j**) to the reference measurements, i.e. mock 2 h, 24 h, and 48 h. ICAM-1—green; glycocalyx (HS)—yellow, vWF—red. Experiments were performed in at least two repetitions, each in duplicate. Statistics: p values were determined by one-way ANOVA followed by Tukey’s post-hoc test, prepared in Origin software. The statistical significance was marked as: (*): p < 0.05, (**): p < 5E − 3, (***): p < 5E − 6.

To address this question, we performed fluorescence tests using selected markers of endothelial function. The results of the qualitative and quantitative analysis are shown in Fig. 4. In particular, we assessed the mitochondrial homeostasis markers like expression of the anti-apoptotic protein Bcl-2 level and mitochondrial oxidative stress (Fig. 4a–d), as well as markers of endothelial dysfunction (Fig. 4e–j). Figure 4a,b present the results of mitochondrial homeostasis markers showing an increase of Bcl-2 protein content with the increase of p.i time. Bcl-2 proteins, known as antiapoptotic proteins, confer cellular resistance to mitochondrial apoptosis and attenuate mitochondrial oxidative stress^39^. As shown (Fig. 4a–d) in infected HPAECs cells, a slight increase in mitochondrial reactive oxygen species was observed (Fig. 4c,d**)**, which in turn we associate with an increase in Bcl-2 activity. Our result shows that after infection with the SARS-CoV-2 virus, a defense reaction develops in endothelial cells, protecting the cells against apoptosis.

The next panel presenting the endothelial dysfunction markers (Fig. 4e–j) has shown that after infection with variant B.1.13 (Wuhan) of SARS-CoV-2 virus in HPAECs develop an inflammatory response. The both qualitative (Fig. 4e,g,i) and quantitative (Fig. 4f,h,j) analysis, indicate a gradual increase in the number of ICAM-1 molecules, a reduction in the glycocalyx layer (heparan sulfate) associated with the rapid increase in the von Willebrand factor (vWF) were observed. An increase in the activity of ICAM-1 adhesion molecules (Fig. 4e,f), a standard marker of endothelial inflammation, indicates an increase in the adhesion of circulating leukocytes to the endothelium and a loss of integrity of the endothelial barrier. At the same time, the glycocalyx is reduced (Fig. 4g,h). The structure of this sugar-rich layer protects the endothelial cells against the adhesion of circulating blood cells^40^ and viruses^25^. Its reduction enhances thrombosis and pro-thrombotic complications in blood vessels. In our work, we also noticed that glycocalyx reduction increases the expression of the vWF (Fig. 4i,j), which further promotes the development of vascular dysfunction and thrombosis.

### Impact of SARS-CoV-2 variants: B.1.1.7 (Alpha), B.1.351 (Beta) and B.1.617.2 (Delta) on the nanomechanical properties of HPAECs

To complete the knowledge about the elasticity changes of endothelial cells caused by the SARS-CoV-2 virus, we performed measurements for three variants: Alpha, Beta, Delta. The previous paragraphs showed a complete analysis of elasticity, cytoskeleton changes, and dysfunction markers for HPAECs after infection with the B.1.13 variant of SARS-CoV-2 virus.

**Fig. 5:**
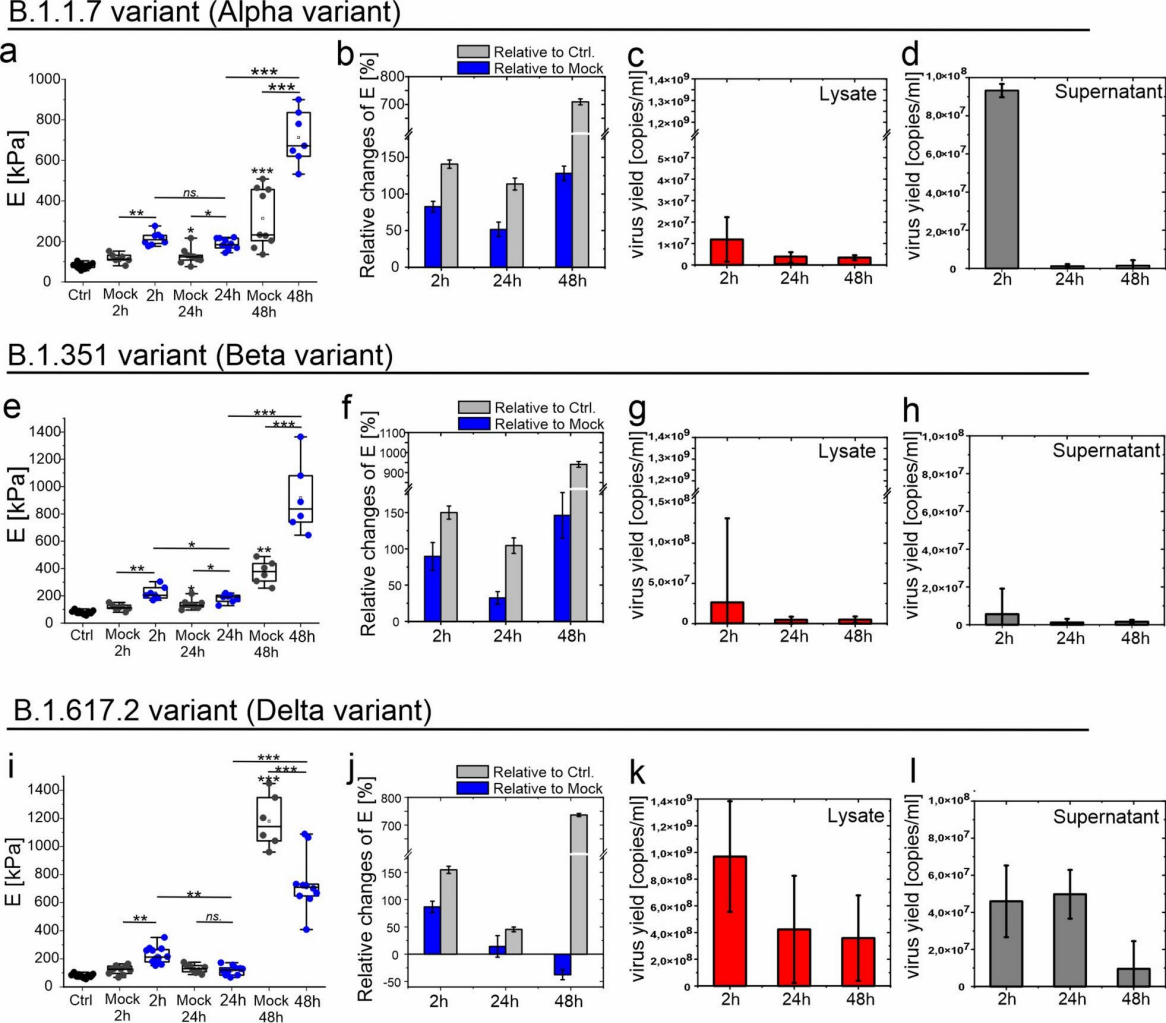
Post-infection changes of HPAECs elasticity and virus replication measured for HPAECs infected with SARS-CoV-2 variants. (**a**–**d**) *Alpha variant (B.1.1.7)*: (**a**) Box-plot depicts the quantitative analysis of elastic moduli (E) calculated for HPAECs after 2 h, 24 h, and 48 h of p.i., reference mock-inoculated HPAECs and non-infected control HPAECs (Ctrl). All measurements were performed for fixed cells. Each point in the box plot represents the mean value of E calculated for a single cell. (**b**) The relative changes of E of infected cells relative to mock (blue) and relative to Ctrl (gray). (**c**, **d**) The graphs show the number of virus RNA copies present in the cells lysate and supernatants collected from HPAECs infected with SARS-CoV-2 variant Alpha at 5000 TCID_50_/ml and collected at 2, 24, and 48 h post-infection. The results are presented as average values with standard deviations (error bars). (**e**–**h**) *Beta variant (B.1.351)*: (**e**) Box-plot of E calculated for HPAECs infected with SARS-CoV-2 Beta variant, mock-inoculated HPAECs and non-infected HPAECs (Ctrl). (**f**) The relative changes of E of infected cells to mock (blue) and relative to Ctrl (gray). (**g**, **h**) The graphs show the number of virus RNA copies present in the cell’s lysate and supernatants. (**i**–**l**) *Delta variant (B.1.617.2)*: (**i**) Box-plot of E calculated for HPAECs infected with SARS-CoV-2 Delta variant, mock-inoculated HPAECs and non-infected HPAECs (Ctrl). (**j**) The relative changes of E of infected cells relative to mock (blue) and relative to Ctrl (gray). (**k**, **l**) The graphs show the number of virus RNA copies present in the cell’s lysate and supernatants. Experiments were performed in at least two repetitions. Statistics: p values were determined by one-way ANOVA followed by Tukey’s post-hoc test, with significance marked as (*): p < 0.05; (**): p < 5E − 3; (***): p < 5E − 6. The figure was created with OriginPro2022. For increased clarity the scale on (**a**) is different than for (**e**, **i**).

In this section, we present changes in the *E* values and analysis of virus replication in HPAECs infected by subsequent variants. Obtained nanomechanical results for infected cells were compared both to the data obtained for the mock-inoculated cells as well as for non-infected control cells (Ctrl). This is because the values of *E* of mock-inoculated cells change in subsequent time points, especially after 48 h p.i. The results are presented in Fig. 5.

At first, we tested the HPAECs infected with SARS-CoV-2 Alpha variant (Fig. 5a–d). The obtained results showed a similar nature of elasticity changes as in the case of the B.1.13 variant (Fig. 5a,b). The relative changes of *E* (Fig. 5b) calculated in relation to mock-inoculated cells and non-infected cells depict a similar course of changes, showing significant loss of elasticity of HPAECs infected with Alpha variant. However, RT-qPCR studies showed no virus replication in infected HPAECs (Fig. 5c,d).

For variant Beta, after infection, HPAECs were significant less elastic relative to mock and Ctrl (Fig. 5e,f). However, for 24 h p.i time, the elasticity changes are less significant than for 2 h p.i. The most significant decrease of elasticity in HPAECs infected with SARS-CoV-2 Beta variant is noted after 48 h p.i. Similarly, to the Alpha variant, no virus replication was observed in HPAECs (Fig. 5g,h).

The last tested variant of the SARS-CoV-2 virus was the Delta variant. Results of the elasticity changes in HPAECs infected with the Delta variant showed a completely different cell response compared to the previous variants. The reason for this difference seems to be the large variability of the *E* values obtained for mock-inoculated cells (Fig. 5i). Therefore, a significant decrease of elasticity compared to mock was obtained solely for HPAECs after 2 h p.i. For 24 h p.i. the change in HPAECs selasticity is insignificant in relation to mock, while, after 48 h p.i., a significant decrease in *E* values was observed in relation to mock (Fig. 5j). However, in the case of HPAECs infected with SARS-CoV-2 Deta variant, the relative changes of *E* calculated in relation to the non-infected cells show significant loss of elasticity of HPAECs at 48 h after infection. In mock-inoculated group, HPAECs were treated with the same conditions and medium as the infected group, except they were not exposed to the virus. The mock medium, collected from mock-inoculated Vero cells, could contain factors sensitizing HPAECs. Mock- infected cells is a reference group designed to evaluate the effects of viral infection on cells or organisms. In a mock-infected group, cells were treated under the same conditions and reagents as the infected group, except that they are not exposed to the virus. Therefore, the medium in the mock group may contain factors produced by VERO cells (in which viruses were reproduced), which in turn can affect the elasticity of endothelial cells. Therefore, the values of *E* results obtained for endothelial cells infected with SARS-CoV-2 variants were also related to the results of the control group in which cells were cultured without medium change.

Analyzing the RT-qPCR result for HPAECs cells infected with the Delta variant of the SARS-CoV-2 virus (Fig. 5k,l), an increase of virus yield in the lysate was observed (10^9^ copies/ml) compared to the Alpha (10^7^ copies/ml, Fig. 5c) and Beta (< 10^7^ copies/ml, Fig. 5g). Proportionally, for subsequent p.i. times, the virus yield in supernatant looks higher than for the Alpha (Fig. 5d) and Beta (Fig. 5h) variants. However, since the amount of virus in the supernatant is low, it cannot be concluded that virus replication occurs in HPAECs. We assume that an increase in the amount of virus in the cell lysate means increased efficiency of virus entry into the cell.

## Discussion

In this work, we have shown the impact of SARS-CoV-2 on the elasticity of epithelial and endothelial cells. In particular, we have shown the correlation between the modification of cellular elasticity and the effectiveness of viral infection. 

Effective infection in A549^+/+^ cells is followed by a significant two-phase change in cellular elasticity: 2 h after infection, cells lose elasticity, while for longer times (24 h and 48 h p.i.) a significant reverse effect is observed.

Moreover, the changes in cell elasticity correlates with increased viral yield in lysate and supernatant. In the case of endothelial cells, we did not observe efficient virus replication, which confirms the reports in the literature of abortive infection of the endothelium by the SARS-CoV-2 virus^22^. However, in our work, we focused on the nanomechanical aspect, proving that the endothelium infected with the SARS-CoV-2 virus loses elasticity significantly.

We postulate that an decrease in cellular elasticity is the early symptom of viral infection. Literature data indicate that a change in endothelial elasticity is a specific biomarker of endothelial function^41^. Endothelial cells are one of the main players in maintaining vascular homeostasis with the ability to act in both sensory and effector capacities^42^. The endothelial elasticity is an important part of the mechanosensitivity mechanism of endothelium which regulates the blood pressure. This is due to the correlation between the nanomechanical properties of cells and the NO production by eNOS, as proposed by Fels et al.^43^ Stiff cells with polymerized actin fibers, resulting in weakened mechanosensitivity, are unable to produce NO, while soft cells overproduce it. Therefore, alterations of endothelial cell elasticity play a notable role in the pathogenesis of a broad spectrum of human diseases including hypertension, stroke, heart disease, diabetes, tumor growth, and metastasis. In our work, we have shown that changes in endothelial elasticity also occur in viral infection and are induced by direct contact of the virus with the endothelium. The loss of endothelial elasticity caused by SARS-CoV-2 virus infection disrupts the endothelial mechanism of blood flow regulation and at the same time amplifies the development of inflammation. This result is particularly important for understanding susceptibility to severe COVID-19 in patients with known viremia. Clinical studies indicate that patients diagnosed with plasma viremia have been more predisposed to vascular and tissue damage related to the severe course of COVID-19^19^.

An decrease in endothelial elasticity is a symptom of a viral infection. However, the question remains about the reason for the lack of virus replication in endothelial cells. In our work, in addition to nanomechanical measurements, we focused on the analysis of some cellular cytoskeleton proteins. In general, cellular cytoskeletal proteins play an important role in viral infection of a cell. Some viruses recruit cytoskeletal proteins from the host cell for intracellular trafficking to move around in the cytoplasm much more quickly than could be accomplished by diffusion alone^33,44–46^. In this work, we focus on the two cytoskeleton proteins—actin and vimentin and also one of the proteins that regulate the structure of the cytoskeleton—RhoA, which have an impact on the nanomechanical properties of cells and contributes to the life cycles of virus.

First, actin forms filaments that provide cells with mechanical support and contribute to biological processes such as mechanosensitivity, internalizing membrane vesicles, and cellular movement and communication^47^. In endothelial cells, in addition to the above functions, actin is responsible for maintaining the impermeability of the endothelial barrier^48^. Reorganization of the actin cytoskeleton is strongly related to changes in the nanomechanical properties of cells and occurs under the influence of various pathological factors^49^. The SARS-CoV-2 virus also uses actin in its replication cycle, hijacking the actin fibers network for moving to replication sites. In this work, we have shown that infection caused by the SARS-CoV-2 virus significantly changes the actin cytoskeleton.

In the case of A549^+/+^ cells, these changes lead to a decrease in the number of actin filaments for longer p.i. times. This result is consistent with previously published data^50^ indicating that efficient replication in A549^+/+^ cells reduces the number of F-actin filaments. Simultaneously, after 2 h p.it the significant increase in the F-actin content in A549^+/+^ cells shown in our work indicates the contribution of actin filaments in the movement of the virus in the cytoplasm.

For endothelial cells, the results obtained indicate a significant increase in F-actin polymerization stimulated by SARS-CoV-2 virus infection. Although for 2 h p.i. in HPAECs the actin content increases, similarly to A549^+/+^, for longer times p.i. the actin content also remains at a high level. This result correlates with the measurement of elasticity and, at the same time, indicates the progression of endothelial cell dysfunction and an increase in the permeability of the endothelial barrier. We speculate that actin polymerization occurring in HPAECs is one of the reasons for the inhibition of virus replication. Additionally, in HPAECs infected with SARS-CoV-2 viruses, the RhoA protein content increases, confirming that a cellular response mechanism related to actin polymerization is activated in endothelial cells. RhoA proteins are responsible for actin polymerization, which in consequence leads to increased cellular stiffening. Importantly, in endothelial cells, an increase in the level of RhoA protein unbalances the production of vasodilating and vasoconstricting substances leading to the development of endothelial dysfunction^51^.

The other cytoskeleton protein strongly related to virus biology is vimentin, which is an intermediate filament cytoskeletal component that plays important roles in the regulation of cellular functions such as migration, response to inflammation, and immunity. Vimentin forms a dynamic, and elastic network surrounding the nucleus and it spreads radially to the cell membrane. Importantly, vimentin plays essential roles in coordinating intracellular signaling pathways, particularly, in endothelial cells vimentin modulates the production of NO as well as regulating the endothelial barrier function^52^. In the context of virus infection, vimentin plays an important role in virus entry and replication. Arrindell et al.^53^ demonstrated a direct interaction between the SARS-CoV-2 spike protein, ACE2, and vimentin in epithelial cells. In this cell, cell surface vimentin works as a coreceptor for SARS-CoV-2 viruses and therefore increases viral entry and cytopathogenic effects. Our results confirm this effect showing a significant increase in the vimentin content in A549^+/+^ cells correlated with effective replication. Interestingly, for HPAECs the vimentin content decreases, which may be a clue to explain the nonproductive SARS-CoV-2 infection of endothelial cells. As shown, there is a relation between vimentin and RhoA activity^54^. Vimentin depletion promotes RhoA activity and actin stress fiber assembly. In our work, we showed that there is an anti-correlation between RhoA and vimentin in HPAECs. The increase in RhoA activity, resulting in polymerization and increased stiffness, correlates with a decrease in vimentin content in HPAECs. In this context, it can be concluded that the interaction of these two cytoskeletal proteins may influence the effectiveness of endothelial cells viral infection.

**Fig. 6:**
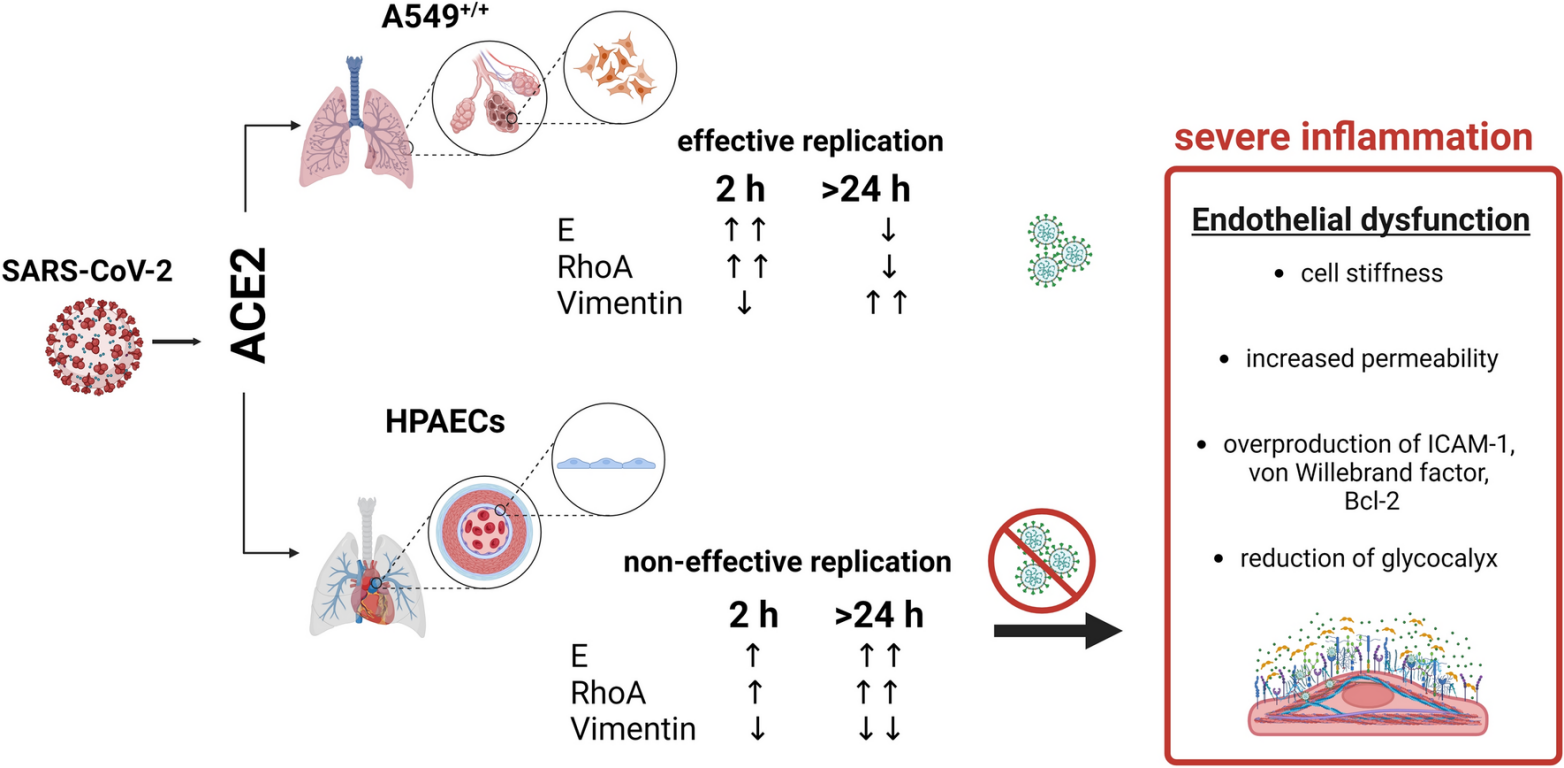
Proposed model of the course of SARS-CoV-2 infection in A549^+/+^ cells and HPAECs. Graphical summary of the results obtained indicating differences in the course of the SARS-CoV-2 infection process in epithelial cells (A549^+/+^) and endothelial cells along with the dysfunctions occurring in HPAECs. The figure was created with BioRender.com.

ACE2 is the main receptor for the SARS coronaviruses family, which regulates the entry of those viruses into cells. In the case of A549^+/+^ cells, the overexpression of ACE2 and TMPRSS indisputably increases the effectiveness of the infection and confirms the significant contribution of these receptors to epithelial infection by SARS-CoV-2. However, for endothelial cells, abortive infection may suggest the participation of other receptors, less specific. In the cardiac system, as shown by Clarke et al.^55^ the ACE2 binds to integrin subunits that affect integrin-induced cell signaling. Cooperation between ACE2 and the integrin receptors could explain endothelial resistance to effective SARS-CoV-2 infection. Integrins play an important role in regulating cellular proliferation, migration, inflammation, and apoptosis^56^. The mechanism of integrin action is related to the expression of RhoA proteins, which are the main transducer of signals from plasma membrane receptors^44,50,57^. RhoA proteins mediate various cellular processes, including actin polymerization, stress fiber formation, cell contraction, and cell adhesion to the associated extracellular matrix. Furthermore, RhoA protein expression is correlated with an increase in Bcl-2 protein activity in HPAECs infected with SARS-CoV-2 (Figs. 3e,g, 4a,b). The published data indicate that the expression of the RhoA protein in endothelial cells blocks the mitochondrial apoptosis pathway due to Bcl-2 protein activation^58^. Bcl-2 also acts as an antioxidant in endothelial cells. The increase in Bcl-2 in infected HPAECs protects the endothelial cells against apoptosis and DNA damage^59–62^. Based on reports from the literature and our data, we assume that SARS-CoV-2 viruses bind to HPAECs by ACE2 or integrins, triggering the RhoA-dependent signaling pathway, which leads to actin polymerization and an increase in cell stiffness and permeability of the endothelial layer^37^. Moreover, as we have shown, the direct exposure of HPAECs on SARS-CoV-2 leads to the increase in the inflammation markers like ICAMs level, glycocalyx reduction, and increase of vWF factor (Fig. 4). The glycocalyx layer plays an important role in the maintenance of selective permeability of the endothelial barrier, modulates leukocyte adhesion, and influences the antithrombotic potential of the endothelial layer. Furthermore, loss of endothelial glycocalyx influences cytoskeleton changes and vice versa^28,63^. The reduction of the glycocalyx layer increases the adhesion of circulating cells and molecules to endothelial cells, therefore, increasing the probability of thrombus formation^64^. The obtained results were presented graphically as a proposed model for the course of SARS-CoV-2 infection in endothelial cells (Fig. 6).

Finally, we observed differences in the endothelial response to infection with SARS-CoV-2 variants (Fig. 5). We showed that all tested SARS-CoV-2 variants (Wuhan, Alpha, Beta and Delta) caused abortive infection in HPAECs. However, the differences in endothelial elasticity depending on the time post infection were noticed. An increase of *E* value was observed for all variants after 2 h p.i., and this trend continued in subsequent periods for the Alpha and Beta variants. In contrast, a decrease of *E* value over time was observed for the Delta variant relative to mock According to literature reports, adaptive mutations of SARS-CoV-2 altered the virus’s pathogenic potential which is associated with increased transmissibility and severity of COVID-19 infection^11,65,66^. In this paper we have shown that changes in endothelial cell elasticity in the response to SARS-CoV-2 virus infection also depend on the virus variant, which in turn may translate into the pathophysiology of COVID-19 development and thus the consequences of infection.

Since the COVID-19 pandemic, research and clinical evidence reveals that manifestations of endothelial dysfunction are widely observed in COVID-19 as well as contributes to the induction of post-infectious vascular complications. However, it is debatable, whether endothelial dysfunction are caused by direct infection of the endothelium or are indirectly by systemic inflammation. The results presented in this paper show for the first time that SARS-CoV-2 infection directly changes endothelial elasticity and causes cytoskeletal reorganization which is related to the development of endothelial dysfunction. This may contribute to a better understanding of the development of vascular complications in COVID-19 as well as the discovery of new directions of therapy.

## Methods

### Cells and viruses

A549 (*Homo sapiens*; lung epithelial cells; ATCC CCL-185) expressing ACE2 and TMPRSS2 (A549^+/+^) was performed according to the standard method used lentivirus approach^67^. Cells were maintained in Dulbecco-modified Eagle’s medium (DMEM, high glucose, ThermoFisher Scientific, Warszawa, Poland) supplemented with 5% heat-inactivated fetal bovine serum (FBS, ThermoFisher Scientific, Poland). The medium was also complemented with penicillin (100 U ml^−1^, ThermoFisher Scientific, Warszawa, Poland) and streptomycin (100 μg ml^−1^, ThermoFisher Scientific, Warszawa, Poland). Furthermore, blasticidin S (10 μg ml^−1^, Sigma-Aldrich, St. Louis, MO, USA) and puromycin (0.5 μg ml^−1^, Sigma-Aldrich, St. Louis, MO, USA) were added to the medium to maintain the expression of ACE2 and TMPRSS2. Vero cells (*Cercopithecus aethiops*; kidney epithelial; ATCC CCL-81) cells were maintained in DMEM supplemented with 3% FBS, 100 U/ml penicillin, and 100 μg ml^−1^ streptomycin. Cells were cultured at 37 °C in a humid atmosphere containing 5% CO_2_. Every two weeks, cells were routinely tested for mycoplasma contamination. Primary Human Pulmonary Artery Endothelial Cells (HPAEC, ATCC PCS-100-022) were grown in Vascular Cell Basal Medium (Cat. No. PCS-100-030, ATCC), supplemented with Endothelial Cell Growth Kit-VEGF (Cat. No. PCS-100-041, ATCC). The medium was also completed with penicillin and streptomycin (10 mg ml^−1^, Sigma-Aldrich, St. Louis, MO, USA).

The SARS-CoV-2 strain isolated in house was used as a reference. The variant (B.1.13) is designated hCoV-19/Poland/PL_P7/2020 (GISAID accession code: EPI_ISL_428930). The Delta variant (B.1.617.2) was isolated from a sample obtained in May 2021 in the Czech Republic and is designated hCoV-19/Czech Republic/NRL_7102/2021 (GISAID accession code: EPI_ISL_2357738). The Alpha variant (B.1.1.7) was purchased from EVAg (Ref-SKU: 012V-04194), SARS-CoV-2, hCoV-19/Sweden/20-53840/2020. The Beta variant (B.1.351) was purchased from EVAg (Ref-SKU: 012V-04195), SARS-CoV-2/hCoV-19/Sweden/21-51217/2021.

All SARS-CoV-2 stocks were generated by infecting of Vero cell monolayer. The cells were incubated at 37 °C under 5% CO_2_. The virus-containing medium was collected on day 2 post-infection (p.i.), aliquoted, and stored at − 80 °C. Control samples from mock-inoculated cells were prepared in the same manner. Virus yields were assessed by titration on fully confluent cell layers in 96-well plates, according to the method of Reed and Muench. Plates were incubated at 37 °C, and the cytopathic effect (CPE) was scored by observation through an inverted microscope.

### Viral infection

A549^+/+^ and HPAEC cultures were seeded in a culture medium on 96-well plates (TPP, Trasadingen, Switzerland) 2 days before infection. Subconfluent cell layers were infected with SARS-CoV-2 viruses at 1600 50% tissue culture infectious dose (TCID_50_)/ml. After 2 h of incubation at 37 °C, cells were rinsed twice with PBS, and a fresh medium was added. The infection was carried out for the next 48 h; supernatants and cells were collected after 2 h, 24 h, and 48 h p.i.

### Isolation of nucleic acids, reverse transcription, and quantitative PCR

A viral DNA/RNA kit (A&A Biotechnology, Gdansk, Poland) was used for nucleic acid isolation from the cell culture supernatants and cells. RNA was isolated following the manufacturer’s instructions. Viral RNA was quantified by the usage of quantitative PCR coupled with reverse transcription (RT-qPCR) (GoTaq Probe 1-Step RT-qPCR System, Promega, Poland), with CFX96 Touch real-time PCR detection system (Bio-Rad, Munich, Germany). The reaction was carried out in the presence of the primers and probe (Fwd: CAC ATT GGC ACC CGC AAT C; Rev: GAG GAA CGA GAA GAG GCT TG; probe: 6FAM-ACT TCC TCA AGG AAC AAC ATT GCC A-BHQ-1). The heating scheme was as follows: 15 min at 45 °C and 2 min at 95 °C, followed by 40 cycles of 15 s at 95 °C and 1 min at 56 °C. To assess the copy number of the N gene, standards were prepared. The PCR product was amplified and cloned into pTZ57R/T plasmids using an InsTAclone PCR cloning kit (Thermo Scientific). The resulting plasmid was linearized, and its concentration was assessed with a NanoDrop™ 2000 spectrophotometer (Thermo Fisher Scientific, Waltham, MA, USA); the number of copies was deducted based on the Avogadro constant. The obtained standards were serially diluted and used as input for RT-qPCR.

### Detection of SARS-CoV-2 N sg mRNA

Total nucleic acids were isolated from the virus- or mock-inoculated cells with Viral DNA/RNA Kit (A&A Biotechnology), following the protocol provided by the manufacturer. The TURBO DNase (Thermo Fisher Scientific, Poland) was added to the samples to remove the DNA contamination; the reaction was carried out for 15 min at 37 °C, and subsequently, the enzyme was inactivated by 10 min incubation at 75 °C in the presence of 10 mM EDTA (Thermo Fisher Scientific, Poland). Reverse transcription was performed using a high-capacity cDNA reverse transcription kit (Thermo Fisher Scientific, Poland), following the manufacturer’s instructions. Viral cDNA was amplified in a 20 µl reaction mixture containing 1 × Dream Taq Green PCR master mix (Thermo Fisher Scientific), and primers (500 nM each). The following primers were used to amplify SARS-CoV-2 subgenomic mRNA (sg mRNA): forward primer TAT ACC TTC CCA GGT AAC AAA CCA; reverse primer—first PCR reaction GTA GCT CTT CGG TAG TAG CCA AT; reverse primer—second PCR reaction TCT TCC TTG CCA TGT TGA GTG A. The conditions were as follows: 3 min at 95 °C, 35 cycles (30 cycles for 2nd PCR) of 30 s at 95 °C, 30 s at 55 °C, and 20 s at 72 °C, followed by 5 min at 72 °C and 10 min at 4 °C. The PCR products were run on 1% agarose gels (1 × Tris–acetate EDTA [TAE] buffer) and analyzed in the dedicated imaging software (Thermo Fisher Scientific).

### Cell preparation for AFM and confocal imaging

HPAECs and A549^+/+^ cells were seeded on a glass coverslip 48 h before the infection. Subconfluent cells were infected with all above-mentioned SARS-CoV-2 variants and after 2 h washed with PBS; the medium was refreshed. The infection was carried out for 2 h, 24 h, and 48 h, whereupon cells were fixed for 1 h with 3.7% paraformaldehyde (PFA) buffered with PHEM.

### Atomic force microscopy measurements

All measurements were carried out using fixed cells in Hanks’ Balanced Salt Solution (H8264, Sigma-Aldrich) as a measuring medium. The AFM nanoindentation experiments were made for fixed cells, according to the procedure described in the paper by Targosz-Korecka et. al.^36^ All samples were mounted into a liquid cell (BioCell, JPK Instruments) under a stable temperature set at 37 °C. The measurement was carried out using NanoWizard 3 NanoScience AFM (JPK Instruments). HPAEC and A549^+/+^ cell AFM imaging was performed using pyramidal-shaped Pt-Ir coated cantilevers (SCM-PIC-V2, Bruker) with a nominal spring constant of 0.1 N/m. Images (256 × 256 pixels) were obtained at scan sizes of 4 μm × 4 μm, 20 μm × 20 μm (for B.1.13 variant) and 30 μm × 30 μm (for B.1.1.7, B.1.351 and B.1.617.2 variants). Topographical images were acquired with chosen force-distance (FD)-based imaging mode (QI; JPK Instruments), allowing for high-resolution imaging of fixed cells. In this method, a single FD-curve is measured in every pixel point of the image and translated from the selected trigger force into the images of cell topography. The loading force varied from 0.7 to 1.2 nN and was adjusted to obtain a clear contrast of the cell surface.

To determine the cell elastic modulus *E* (exclusively refers to the apparent Young’s modulus), the set of force-distance curves was analysis by fitting the Hertz-Sneddon model for a conical indenter^68^. Obtained results have been present in a form of spacial elastic modulus maps (qualitative representation of the data) and as mean elastic modulus value calculated for each measured cells (quantitative analysis in form of box plot). As result, for each measured cells we obtained a topography images correlated with the elasticity map. The images of topography and the elastic modulus were derived using JPK Data Processing Software. Experimental and analysis details are presented in the Supplementary Information.

### Fluorescence staining and imaging

Fixed cells were permeabilized using 0.5% Tween-20 (13 min, room temperature [RT]), and unspecific binding sites were blocked with 5% bovine serum albumin (BSA) in PBS (4 °C, overnight) prior to staining. Cells on coverslips were stained to visualize cellular and viral proteins. All antibodies used for immunostaining are listed in Table 1.

**Table 1:** Reagents and dyes used in the measurements.

Antiobdy	Provider	Catalog number	Dilution	Conditions
Mouse SARS-CoV-2 N protein antibody	Thermo Fisher Scientific	MA5-29981	1:200	2 h, RT
Mouse Anti-ICAM-1/CD54 antibody	Santa Cruz Biotechnology	Sc-8439	1:500	1.5 h, RT
MitoSOX™ Red Mitochondrial Superoxide indicator	Molecular Probes	M36008	1:1000	30 min, RT
Anti-Rho A antibody conjugated with Alexa Fluor 647 dye	Santa Cruz Biotechnology	Sc-48 AF647	1:500	3 h, 4 °C
Bcl-2 monoclonal antibody conjugated with FITC dye	Thermo Fisher Scientific	A18153	1:500	45 min, RT
Recombinant anti-vimentin antibody	Abcam	Ab92573	1:200	O/N, 4 °C
Anti-heparin sulfate primary antibody	Amsbio	370255_S	1:100	O/N, 4 °C
Phalloidin conjugated with Alexa Fluor 647 dye	Thermo Fisher Scientific	A22287	1:400	25 min, RT
Phalloidin conjugated with Alexa Fluor 488 dye	Thermo Fisher Scientific	A12379	1:400	25 min, RT
Secondary antibody conjugated with Alexa Fluor 488 dye	Thermo Fisher Scientific	A-11001	1:400	1 h, RT
Secondary antibody conjugated with Alexa Fluor 555 dye	Thermo Fisher Scientific	A21422	1:200	1 h, RT
Secondary antibody conjugated with Alexa Fluor 546 dye	Thermo Fisher Scientific	A11003	1:500	1 h, RT
vWF antibody	Santa Cruz Biotechnology	Sc-53466	1:500	1 h, RT
m-IgGκ BP-CFL 594	Santa Cruz Biotechnology	Sc-516178	1:500	1 h, RT

After incubating with each antibody, cells were washed thrice with 0.5% Tween-20 in PBS. Finally, the nuclear DNA was stained with 4’, 6-diamidino-2-phenylindole (DAPI, 0.1 mg/ml, Sigma-Aldrich), washed, and mounted on glass slides with Prolong Diamond antifade mountant (P36970, Thermo Fisher Scientific, Poland). Samples were visualized using Zeiss LSM 710 confocal microscope and 40×/1.3 oil objective.

### Statistical analysis

The AFM experiment was performed in two replicates, with 6–12 elasticity maps measured for each data point. Each map contains between 1 and 3 cells. The total number of cells measured for the subsequent data point is summarized in the Supplementary Information. The AFM nanoindentation data were presented in the form of a box-plot. Each point in box-plot graphs represents the mean elasticity modulus (E) calculated for a single elastic maps based on a log-normal distribution fit. The data used to prepare the histogram include only the values measured on the cells, without the data from the background (glass) (see Supplementary Information). Next, the mean values were calculated for each data points and groups (infected cells, mock and Ctrl) in order to compute the relative changes, relative to mock and Ctrl, based on the Eq. (1):

1 $$Relative=\frac{{{I}_{i}^{SARS-CoV-2}}-{I}_{i}^{R}}{{I}_{i}^{R}}\times 100\%$$

where ${I}_{i}^{SARS-CoV-2}$ are the means of elastic modulus for infected cells and ${I}_{i}^{R}$ are the means of elastic modulus mock cells or Ctrl cells after *i* ∈ {2, 24, 48} h of incubation. The absolute error of such measurements was calculated as a total differential of the Relative function.

The fluorescence intensity data were analyzed in ImageJ software. For a single fluorescence image, the intensity was measured as a total value and normalized to the background value and a number of cells. Next, the mean fluorescence value was calculated based on all measured images, with SD as an error. To compare the data, the relative change was calculated based on the Eq. (2):

2 $$Relative=\frac{{{I}_{i}^{SARS-CoV-2}}-{I}_{i}^{Mock}}{{I}_{i}^{Mock}}\times 100\%$$

where ${I}_{i}^{SARS-CoV-2}$ and ${I}_{i}^{Mock}$ are the means s of fluorescence intensity for infected cells and mock cells after i ∈ {2, 24, 48} h of incubation. The absolute error of such measurements was calculated as a total differential of the Relative function.

The statistical significance was tested with a one-way ANOVA (P < 0.05) followed by Tukey’s honest significant difference post-hoc test. All statistical analyses and graphs were prepared in Origin software.

## Supplementary Information

Supplementary Information.

## Data Availability

The datasets used and/or analysed during the current study available from the corresponding author on reasonable request.
