# Supplementary material for: Remodeling of intracellular architecture during SARS-CoV-2 infection of human endothelium
Source: Sci Rep. 2024 Nov 30;14:29784. doi: 10.1038/s41598-024-80351-z (PMC11608320; doi:10.1038/s41598-024-80351-z)
Supplement: Supplementary file 1 — Supplementary Information. [file 41598_2024_80351_MOESM1_ESM.docx]

**Supplementary information**

Remodeling of Intracellular Architecture During SARS-CoV-2 Infection of Human Endothelium.

Agata Kubisiak^1,2^*, Agnieszka Dabrowska^3^*, Pawel Botwina^3^, Patrycja Twardawa^1,2^, Damian Kloska^4,5^, Tomasz Kołodziej^6,7^, Zenon Rajfur^7^, Krzysztof Pyrc^3^, Marta Targosz-Korecka^1^

*^1^Jagiellonian University, Faculty of Physics, Astronomy and Applied Computer Science, M. Smoluchowski Institute of Physics, Department of Physics of Nanostructures and Nanotechnology, Kraków, Poland*

*^2^Jagiellonian University, Doctoral School of Exact and Natural Sciences, Kraków, Poland*

*^3^Jagiellonian University, Malopolska Centre of Biotechnology, Virogenetics Laboratory of Virology, Kraków Poland*

*^4^Jagiellonian University, Faculty of Biochemistry, Biophysics and Biotechnology, Department of Medical Biotechnology, Kraków, Poland*

*^5^Selvita Services, Kraków, Poland*

*6Department of Pharmaceutical Biophysics, Faculty of Pharmacy, Jagiellonian University Medical College, Kraków, Poland*

*^7^Jagiellonian University, Faculty of Physics, Astronomy and Applied Computer Science, M. Smoluchowski Institute of Physics, Department of Molecular and Interfacial Biophysics, Kraków, Poland*

**
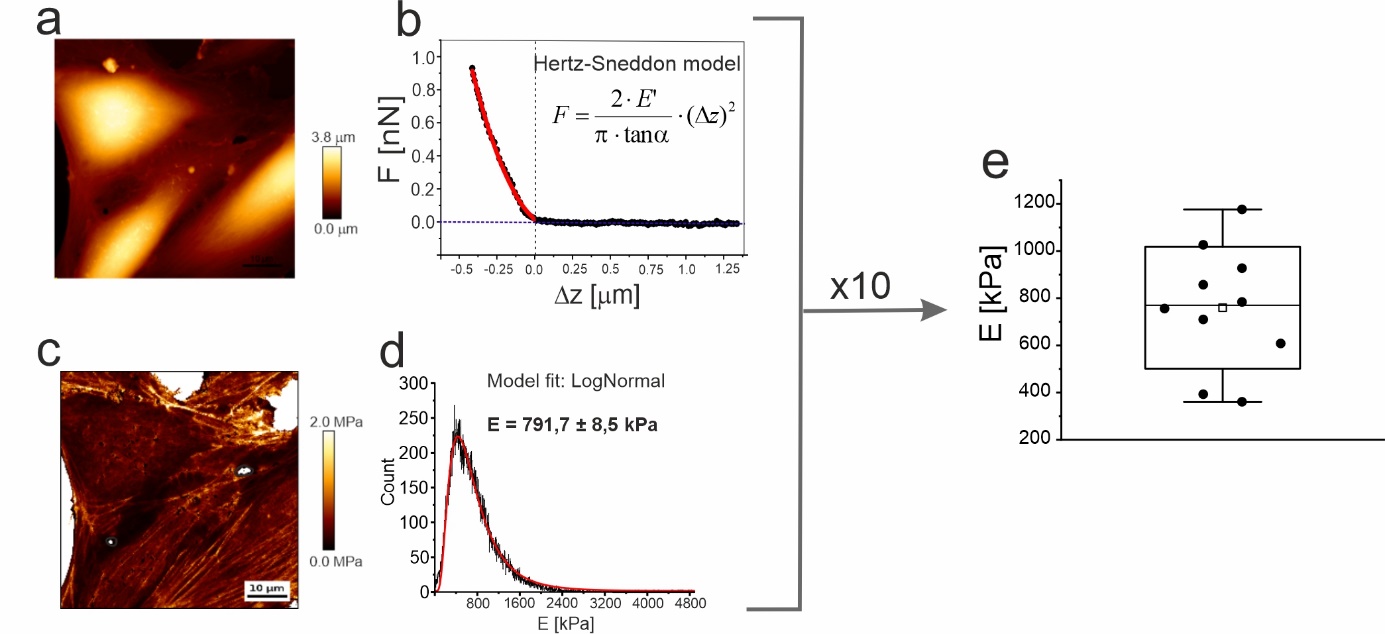
**

**Supplementary Figure 1 Experimental details of the AFM measurements and data analysis**. a) Example of AFM maps measured for cells from selected region of sample. b) An example of the indentation curve recorded for the cell body and the Hertz-Snedon model fit at maximum load. c) Elasticity maps. d) Histogram of the elastic modulus calculated for the cell body (without the white area on the elasticity maps). All procedures were repeated 10 times in two independent experiments. E) Box plots of mean elastic modulus values, each point corresponding to the mean value of E obtained for cells from one map.

Measurements were performed in duplicate. For each test system (i.e. mock and infected cells), 3 samples were prepared, one of which was used for AFM measurements and the other two for fluorescence staining. We used this protocol to ensure complete consistency between AFM results and fluorescence images. We are aware that AFM studies are time consuming and when working with samples prepared with active viruses, we had 24 hours to take all the images. After this time the samples were removed.

For the AFM measurements, approximately 10 maps in two repetition were measured at random locations. The single map contains between 1 and 3 cells. The AFM maps were taken at a resolution of 256x256 points and a single force-distance curve was taken at each point. The Hertz-Sneddon model for the conical probe (α=15^0^) was used to analyse the modulus of elasticity:

$$F = \frac{2\cdot E}{\pi\cdot\tan\alpha} \cdot(\Delta z)^{2}$$

The resulting elastic modulus, *E* (apparent Young’s modulus) was calculated for an approximate Poisson's ratio of 0.5:

$$E = \frac{E_{cell}}{1-\mu_{cell}^{2}}$$

Based on the elasticity map, only the E values from the indentation curves measured for cells were used to create the histogram.

The number of maps and cells on which the analysis was based is summarised in the table below.

**Supplementary Table 1. Summary of studied systems.** n – number of AFM experiments (repetition). m – total number elasticity maps. N – number of cells.

| Cell type | System | Figure | n | m | N |
| --- | --- | --- | --- | --- | --- |
| A549^+/+^ | 2h mock | 2a | 2 | 6 | 11 |
|  | 24h mock | 2a | 2 | 7 | 14 |
|  | 48h mock | 2a | 2 | 7 | 16 |
|  | 2h SARS-CoV-2 (B.1.13) | 2a | 2 | 10 | 18 |
|  | 24h SARS-CoV-2 (B.1.13) | 2a | 2 | 10 | 20 |
|  | 48h SARS-CoV-2 (B.1.13) | 2a | 2 | 10 | 16 |
| HPAEC | 2h mock | 2e | 2 | 6 | 10 |
|  | 24h mock | 2e | 2 | 6 | 13 |
|  | 48h mock | 2e | 2 | 6 | 15 |
|  | 2h SARS-CoV-2 (B.1.13) | 2e | 2 | 10 | 20 |
|  | 24h SARS-CoV-2 (B.1.13) | 2e | 2 | 10 | 16 |
|  | 48h SARS-CoV-2 (B.1.13) | 2e | 2 | 8 | 18 |
| HPAEC | 2h mock | 5a | 2 | 7 | 14 |
|  | 24h mock | 5a | 2 | 9 | 14 |
|  | 48h mock | 5a | 2 | 9 | 18 |
|  | 2h SARS-CoV-2 (B.1.1.7) | 5a | 2 | 8 | 18 |
|  | 24h SARS-CoV-2 (B.1.1.7) | 5a | 2 | 10 | 16 |
|  | 48h SARS-CoV-2 (B.1.1.7) | 5a | 2 | 7 | 14 |
| HPAEC | 2h mock | 5e | 2 | 7 | 12 |
|  | 24h mock | 5e | 2 | 8 | 14 |
|  | 48h mock | 5e | 2 | 6 | 14 |
|  | 2h SARS-CoV-2 (B.1.351) | 5e | 2 | 7 | 15 |
|  | 24h SARS-CoV-2 (B.1.351) | 5e | 2 | 7 | 14 |
|  | 48h SARS-CoV-2 (B.1.351) | 5e | 2 | 6 | 14 |
| HPAEC | 2h mock | 5i | 2 | 10 | 16 |
|  | 24h mock | 5i | 2 | 8 | 14 |
|  | 48h mock | 5i | 2 | 6 | 13 |
|  | 2h SARS-CoV-2 (B.1.617.2) | 5i | 2 | 12 | 24 |
|  | 24h SARS-CoV-2 (B.1.617.2) | 5i | 2 | 10 | 16 |
|  | 48h SARS-CoV-2 (B.1.617.2) | 5i | 2 | 10 | 22 |
